# False Lumen Haemodynamics in Type B Aortic Dissection: An in Vitro Study Using PIV and Patient-Specific Flexible Phantoms

**DOI:** 10.1007/s10439-025-03875-z

**Published:** 2025-11-22

**Authors:** A. Koulogiannis, Q. Li, S. Homer-Vanniasinkam, V. Diaz-Zuccarini, S. Balabani

**Affiliations:** 1 Department of Mechanical Engineering, University College London (UCL), London, UK; 2 UCL Hawkes Institute, University College London (UCL), London, UK; 3 Leeds Teaching Hospitals NHS Trust, Leeds, UK

**Keywords:** Aortic dissection, Flexible phantoms, Particle image velocimetry, False lumen, Haemodynamics, Patient-specific analyses

## Abstract

Aortic dissection (AD) is a catastrophic vascular pathology caused by delamination of the vascular wall and the formation of a false lumen. False lumen haemodynamics is a key determinant of aneurysmal growth, rupture, and thrombosis. Quantifying the haemodynamics in the false lumen can provide markers to predict these events and stratify patient risk. While such metrics can be extracted from numerical simulations or imaging modalities such as 4D flow MR, high-resolution experimental data are needed to validate them. The present study provides an in vitro characterization of the flow inside the false lumen of a type B aortic dissection using a patient-specific flexible phantom and Particle Image Velocimetry. A mock circulatory loop imposing patient-specific flow waveforms at the inlet and outlets of the aortic phantom and a refractive index matching blood analog were employed. Time-resolved measurements of the velocity field in four selected planes of the false lumen were acquired. Compared against our previous work on the same dissection assuming rigid walls, the results demonstrate the impact of wall compliance on the flow in the false lumen. They revealed the generation of a jet during the systolic phase that enters the false lumen through the primary tear and impinging on the opposite wall with high velocity, generating a strong rotational flow therein. During the diastolic phase, a reversal of the flow was observed generating multiple vortical structures both inside the true and false lumen. Haemodynamic markers such as false lumen ejection fraction were calculated and compared with clinical measurements. The results provide an insight on AD haemodynamics and highlight the potential of this in vitro method as a validation tool for simulations, as well as to physically test interventions in vitro.

## Introduction

Aortic dissection (AD) is a complex and highly patient-specific vascular condition in which a tear forms on the aortic wall allowing blood to flow in and create a false lumen (FL). It is associated with pathological changes in haemodynamics and vascular remodeling that are described in great detail in the recent review by Rolf-Pissarczyk et al. [1]. One such change is aneurysmal growth that occurs in most AD patients [2] and may lead to adverse events, such as aortic rupture. These are closely linked to haemodynamic markers such as pressure and wall shear stress indices that cannot be directly measured in the clinic but can be extracted from image-driven, patient-specific numerical simulations [3]. AD growth and intervention decisions for TBAD are currently based on morphological factors such as maximum aortic diameter. However, false lumen flow-derived markers have been found to be better risk predictors to stratify patients compared to morphological parameters [4]. High false lumen antegrade systolic flows accompanied by significant diastolic retrograde flow have been associated with higher risk of complication in an imaging study of 131 ADs. False lumen ejection fraction (FLEF, the ratio between retrograde and antegrade FL flow) has been related to aortic enlargement.

While such markers are not typically assessed in the clinic—due to modalities such as 4D flow MRI not being routinely used in most centers—numerical studies, by our own group [5, 6] and others, have demonstrated the power of haemodynamic simulations for Type B aortic dissections, in predicting growth [3], and in guiding interventions [7]. The need for compliant simulations to capture the wall motion has also been highlighted.

These patient-specific studies rely on routinely acquired, available, and often sparse clinical datasets that necessitate various assumptions to be made. Experiments provide a physical platform to shed light on fundamental aspects of AD haemodynamics, test modeling assumptions, and intervention scenarios. The experimental approaches employed to date to study AD have been extensively reviewed by Rolf-Pissarczyk et al. [1]. Notably, patient-specific experimental studies quantifying AD haemodynamics are relatively scarce. Many in vitro studies [8–11], have utilised idealized AD geometries to study the effect of morphological parameters such as tears on true lumen (TL) and false lumen (FL) haemodynamics (e.g. flow patterns, pressures and intimal flap (IF) motion). These studies have provided significant insight on the complex flow interactions between the two lumens under unsteady pulsating flow, intima flap (IF) motions, and the pressure and flow patterns therein.

Recent studies have leveraged 3D printing methods to manufacture more complex anatomical models of AD and combined those with clinical imaging modalities to study their haemodynamics. For example, Birjiniuk et al. [12] fabricated an AD silicone phantom based on clinical computed tomography (CT) images and demonstrated through 4D PCMR measurements the altered haemodynamics in a dissected aorta as opposed to a normal one. They reported reverse flow indices up to 20% in the proximal false lumen which they attributed to a pumping like action in the false lumen, large IF displacements, and a vortex near the exit tear. In a later study, Birjiniuk et al. [13] showed that intermediate fenestrations between entry and exit tear reduced the flow reversal in the false lumen. Chen et al. [14] employed patient specific silicone phantoms of a normal and a dissected aorta to demonstrate the use of a mock circulatory loop for haemodynamic analysis. Using Doppler ultrasound, they observed negative velocities in the false lumen indicating flow reversal, and flow exchange between the two lumina in the entry/re-entry tears. Morris et al. [15] produced a thin-walled patient-specific silicone phantom of AD with multiple tears and measured the intimal flap displacement, pressure, and flow patterns in both, true and false lumen, using pressure transducers and ultrasound, at various sections along the dissected aorta. IF displacements of 0.5 to 3 mm were reported. Zimmermann et al. [16] performed 4D flow MRI on an in vitro compliant phantom and used the data to test FSI CFD simulations. A new approach to automate patient-specific type B aortic dissection model fabrication was recently demonstrated by Aghilinejad et al. [17]. Using deep-learning-based segmentation for negative mould manufacturing they were able to create physiologically accurate flexible phantoms for MR imaging. However, no flow characterisation was provided, only pressure waveforms were reported. Recently, Mohl et al. [18] demonstrated the use of 3D-printed patient-specific aortic dissection phantoms with a flexible dissection for TEVAR intervention planning. The study demonstrated, through Doppler ultrasound and pressure measurements, the presence of chaotic flow patterns on main entry and re-entry tears as well as the value of their in vitro approach as a surgical training and planning tool.

While clinical modalities are relevant in the context of dissection haemodynamics, their temporal and spatial resolution tend to be limited compared to optical flow diagnostic techniques such as Particle Image Velocimetry (PIV), employed by the fluid mechanics community to probe complex unsteady flows. Such studies are amenable to in vitro vascular haemodynamics and have been demonstrated in the context of many pathologies. They provide a highly controlled environment to quantify vascular flows; however, they also pose additional challenges when applied to complex geometries such as AD, due to the need for optical access. Hence, it is not surprising that PIV studies of patient-specific AD are rare. Our group was the first to apply PIV to study the haemodynamics of a patient-specific AD and compare the results with CFD models for the same patient using rigid phantoms and a highly flexible and tuneable mock circulatory loop [19, 20]).

In the present study, a transparent, flexible, and patient-specific phantom of the same type B dissected aorta is fabricated and its haemodynamics are studied by means of time resolved particle image velocimetry (TR-PIV), under patient-specific conditions. Previous work on this patient geometry indicated that CFD simulations based on rigid wall assumptions fail to predict any flow in the false lumen unlike those assuming compliant wall simulations [5]. This in vitro investigation tests this assumption by *simultaneously* resolving the flow in the false lumen and wall displacement. The measured flow fields allow the extraction of haemodynamic markers such as jet dynamics, vorticity, and ejection fraction that might serve as predictors of aneurysmal growth and thrombus formation. To the best of our knowledge, this is the first study to employ Particle Image Velocimetry (PIV) in a compliant, patient-specific aortic dissection (AD) phantom, enabling simultaneous measurements of both velocity fields and wall deformation. This work advances the state-of-the-art in in vitro AD haemodynamics by demonstrating a highly tunable and physiologically relevant experimental platform to validate computational workflows and provide insights into aortic dissection flow dynamics at a resolution currently unattainable in clinical practice. This can aid the development of risk stratification markers for AD and testing of intervention scenarios, towards personalising AD treatment.

## Experimental Methods

### Patient-Specific AD Model and Phantom Fabrication

The study is based on clinical data for a 77-year-old presenting with a chronic type B AD, acquired at Leeds General Infirmary following an approved protocol. (NHS Health Research Authority, ref: 12/YH/0551; Leeds Teaching Hospitals NHS Trust, ref: 788/RADRES/16). A rich dataset was available for this patient comprising computed tomography (CT) and 2D PC-MRI scans which allowed us to develop a novel moving boundary computational pipeline for compliant simulations [5], as well as a tunable experimental platform for in vitro investigations [19, 21].

The AD geometry was extracted from the CT scans using Simpleware ScanIP (Synopsys Inc., Sunnyvale, CA, USA) as described in [5]. The geometry is characterised by one entry tear only, located around 10 mm from the proximal end of the dissection. After careful examination of the images by the radiologist in charge and the managing clinician, it was established that no other tears were present in this patient.

**Fig. 1:**
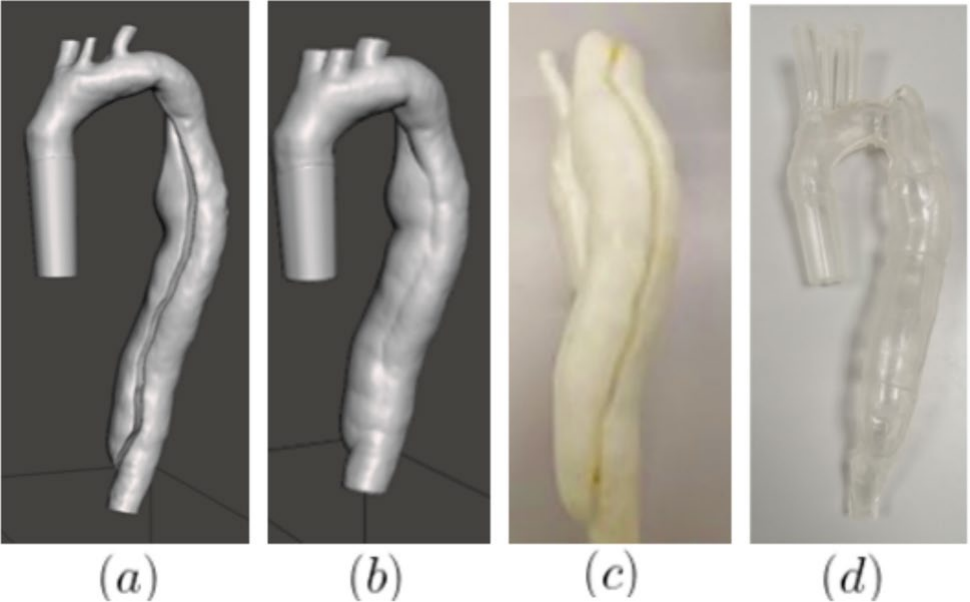
Fabrication process showing the CAD designs of the inner (**a**) and outer mould (**b**), the PVA3D-printed inner mould (**c**), and the final PDMS AD phantom (**d**)

A flexible phantom was manufactured using the lost core technique (see Fig 1) as follows: An inner mould comprising the TL and FL sections as well as an outer mould facilitating the casting process and controlling the wall thickness, were 3D printed using water-soluble polyvinyl alcohol (PVA) (S3 and S5, Ultimaker B.V., Utrecht, Netherlands). The 3D-printed parts were designed with integrated alignment pins ensuring that the inner and outer moulds were properly centered during assembly. To address the surface roughness caused by 3D printing, both parts were refined using a water brushing process partially dissolving the PVA material and smoothing out irregularities. A clear silicone mixture was prepared from Sylgard184 (Dow Corning Corporation, Midland, MI, USA) in a 1:10 ratio of curing agent to polymer. It was degassed in vacuum chamber and poured over the mould in stages to eliminate bubble formation. The phantom was left to cure at room temperature for 24 h and the PVA moulds dissolved in a circulating water bath (Ultimaker B.V., Utrecht, Netherlands).

The phantom was imaged using a microCT scanner in the UCL Mechanical Engineering’s Advanced Characterisation Laboratory for Materials & Manufacturing (ACLMM) (Micro-CT XT H 225, Nikon Co., Tokyo, Japan). The wall thickness of the silicone phantom exhibited some spatial variation along the aortic length, arising from the fabrication method employed. The outer wall thickness was found to be on average 4.3 mm with a standard deviation of 2.4 mm, whereas that of the intimal flap 1.75 mm with a standard deviation of 0.52 mm. While such variability can significantly influence wall deformation predictions in conventional FSI simulations (that assume homogeneous wall properties), this can be accounted for in our modelling approach, which employs a moving boundary method [5] tuned via the wall motion derived from imaging. The Young modulus was measured in a uniaxial testing rig (BT1-FR5.0TN, Zwick Roell Group, Ulm, Germany) using dumbbell-shaped specimens and found to be 0.7 ± 0.03 MPa, which is within the range of values reported for a healthy aorta [22] and close to those utilised for FSI simulations of aortic dissection [23]. The refractive index of the phantom was measured using a gem refractometer and found equal to 1.41.

### Experimental Setup and Particle Image Velocimetry

**Fig. 2:**
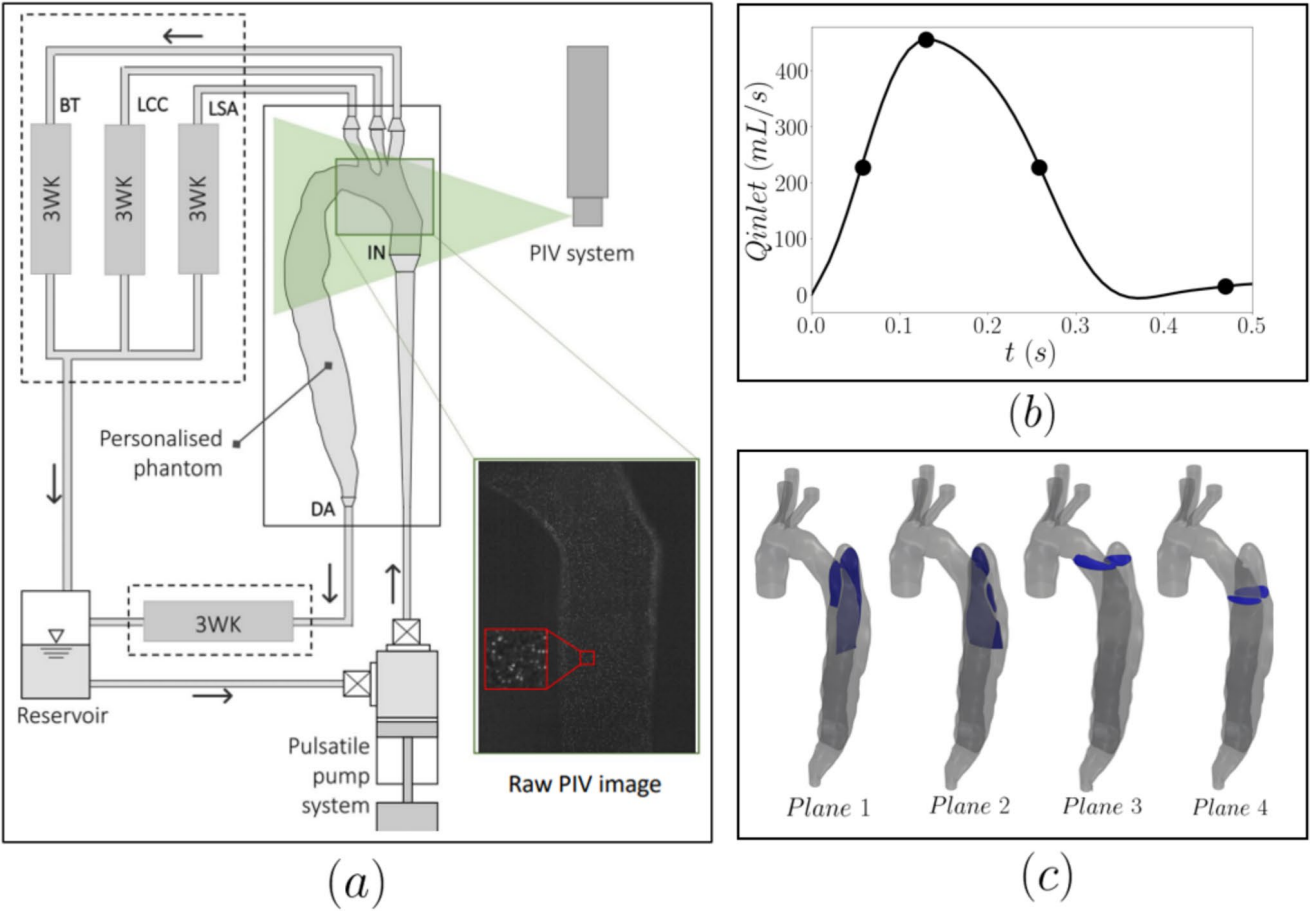
Experimental PIV setup (**a**), imposed flow waveform at the inlet of the phantom indicating the four timepoints selected to visualise the results (**b**), and schematic of the four measurement planes (**c**)

The phantom was mounted on a mock circulatory loop described in detail in Franzetti et al. [20]. The loop (see Fig 2a) comprises a LabView driven piston pump to deliver the pulsatile flow, a chamber mimicking the ventricle and physical Windkessel models at the outlets to replicate the dynamic boundary conditions employed in our computational work, imposing patient-specific waveforms at the inlet and all outlets of the AD. A flow wave obtained from patient PC-MRI data was imposed at the inlet (see Fig 2b) and dynamic (Windkessel) boundary conditions at the outlets [19, 21] to match the patient-specific condition simulated in Bonfanti et al. [5]. The process of tuning the parameters of these Windkessel models is described in Franzetti et al. [20] and Bonfanti et al. [19].

To match the refractive index of the phantom, a water-glycerol-urea solution (as demonstrated by [24] was used in a ratio of 45.64%:28.77%:25.58% by weight and with the following properties: RI = 1.4118, viscosity µ= 3.5*mPa s*, density ρ=1130 *kg/m*^*3*^. The Reynolds number defined by the peak inlet velocity was 6182 and the Womersley number 24.7.

The flow was seeded with Rhodamine B fluorescent polymer particles (20–50 μm, Dantec Dynamics, Skovlunde, Denmark) and illuminated by a 5W continuous wave laser light sheet (Diode-Pumped Solid-State Laser, Laserglow Technologies, Toronto, ON, Canada). A high-speed CMOS camera (Phantom VEO710, AMETEK Inc, Berwyn, PE, USA) equipped with a 550-nm cutoff filter was employed to acquire images for time-resolved PIV (TR-PIV) measurements. A mirror arrangement was used to image cross sections of the TL and FL. Measurements were taken at two selected planes across the false lumen, one at the level of the entry tear and one further below. Two additional vertical planes cutting through the false lumen were also acquired as shown in Fig 2c.

The wall displacement was estimated from the same images. To make the wall visible, a small amount of Rhodamine B (0.0001 g/L) was added to the working fluid so that the quality of the images was not compromised.

A high frame rate, in the range of 3 to 7.5 kHz, was employed to resolve the wall motion and obtain high temporal resolution measurements within each cycle. This was due to the small number of cycles that could be measured without jeopardizing the integrity of the phantom under continuous flow cycling. The number of frames acquired varied between 3000 and 7500 depending on the plane imaged. The images were first masked manually, using the image intensity and the contrast with the wall induced by the Rhodamine B dye, and processed using a cross-correlation algorithm implemented in Dynamic Studio (Dantec Dynamics, Skovlunde, Denmark). Adaptive cross-correlation was used with an initial interrogation window of 8 pixels, a final of 64 pixels and 50% overlap ratio. A 3 iteration-3 x 3 moving average validation filter with a 0.1 acceptance factor followed by a 3 x 3 neighboring vector average filter were applied to the generated vector fields.

The spatial resolution, expressed as pixel-to-millimeter calibration in each plane is as follows: Plane 1: 13.62 pixels/mm, Plane 2: 13.62 pixels/mm, Plane 3: 14.45 pixels/mm, and Plane 4: 13.04 pixels/mm.

Four instances of the cardiac cycle were selected for the visualisation of the vector fields (see Fig 2b). To smoothen the highly resolved results in each cycle, ten consecutive instantaneous vector fields were averaged for each instance. These correspond to less than 0.5% of the cardiac cycle ensuring that the flow variation within the visualized instance is negligible. The resulting velocities were then averaged over 3 cardiac cycles. The average cycle to cycle variation was about 7.5%. The results were visualised in terms of velocity magnitude and helicity which was calculated as follows:

1 $$H\left( t \right) = \mathop \smallint \limits_{V}^{ } u\left( {x,t} \right) \cdot \omega_{z} \left( {x,t} \right)dV,$$

where $u\left( {x,t} \right)$ is the velocity field and ${\omega }_{z}\left(x ,t\right)$ is the z component of vorticity.

It should be noted that due to the different ranges of velocity and helicity magnitude between the true and false lumen, as well as the selected phases in the cardiac cycle, distributions are shown in absolute rather than normalised values.

## Results

**Fig. 3:**
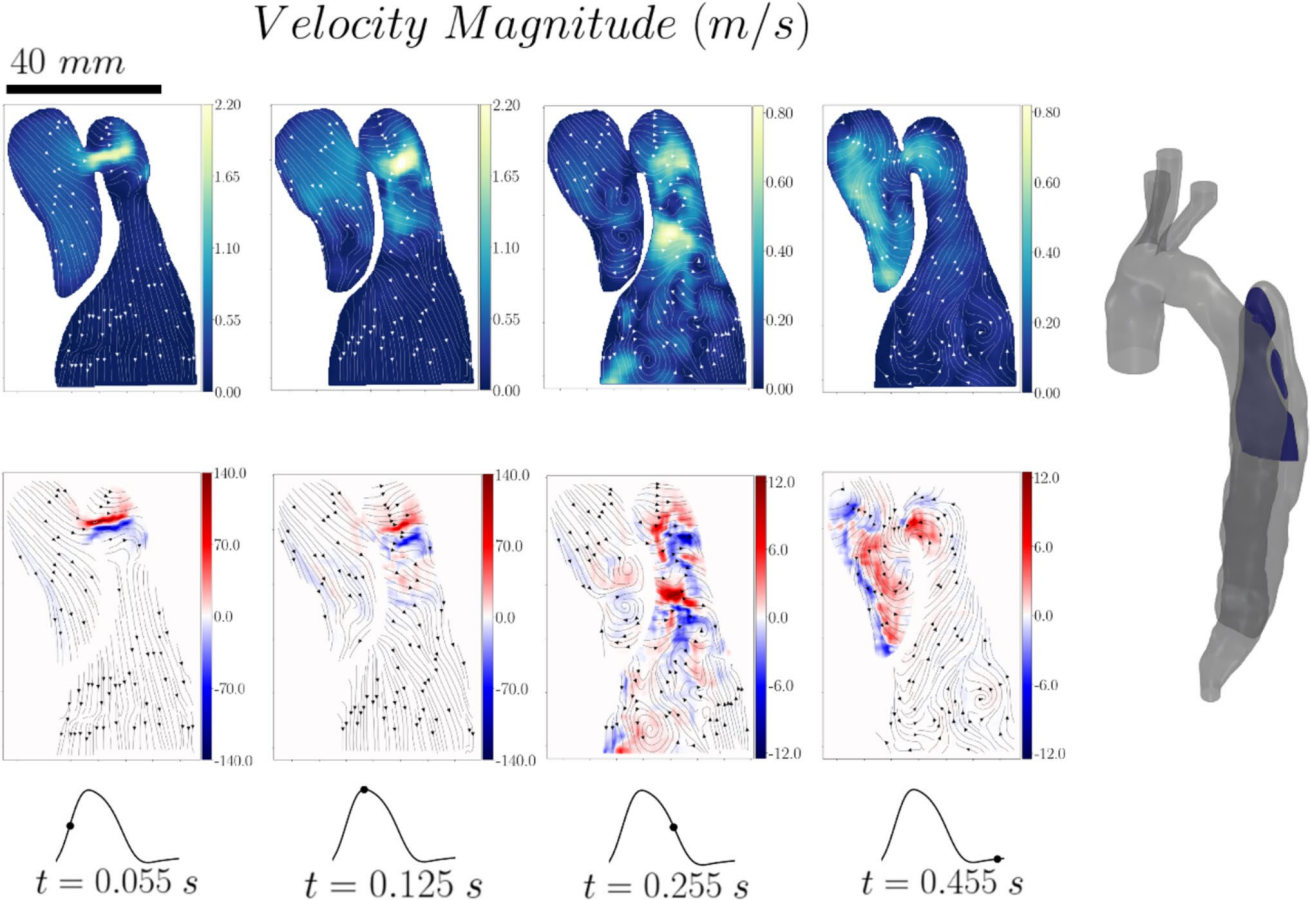
Velocity magnitude and helicity contour maps with superimposed streamlines for Plane 1

Velocity and helicity contour maps with superimposed streamlines in a vertical plane through the proximal end of the dissection (Plane 1) are shown in Fig 3. At mid-systole (*t* = 0.055 s), a high-velocity jet enters the false lumen through the primary entry tear. Antegrade flow can be observed in the rest of the domain, including both the true and false lumen; it is uniform, as evidenced by the parallel streamlines and exhibits low-velocity magnitudes. At peak systole (*t* = 0.125 s), the jet penetrates further into the false lumen and impinges on the opposite wall. Its energy starts dissipating as the diastolic phase begins (*t* = 0.255 s) and the flow propagates further into the false lumen and away from the entry tear. More chaotic flow can be seen, characterised by the presence of small vortical structures. Some vortical structures can also be seen in the true lumen below the entry tear. As the diastolic phase progresses (*t* = 0.455 s), the flow reverses and re-enters the true lumen. High velocities are generated therein, and the flow becomes more chaotic with many vortical structures appearing; also evidenced by the high helicity values.

**Fig. 4:**
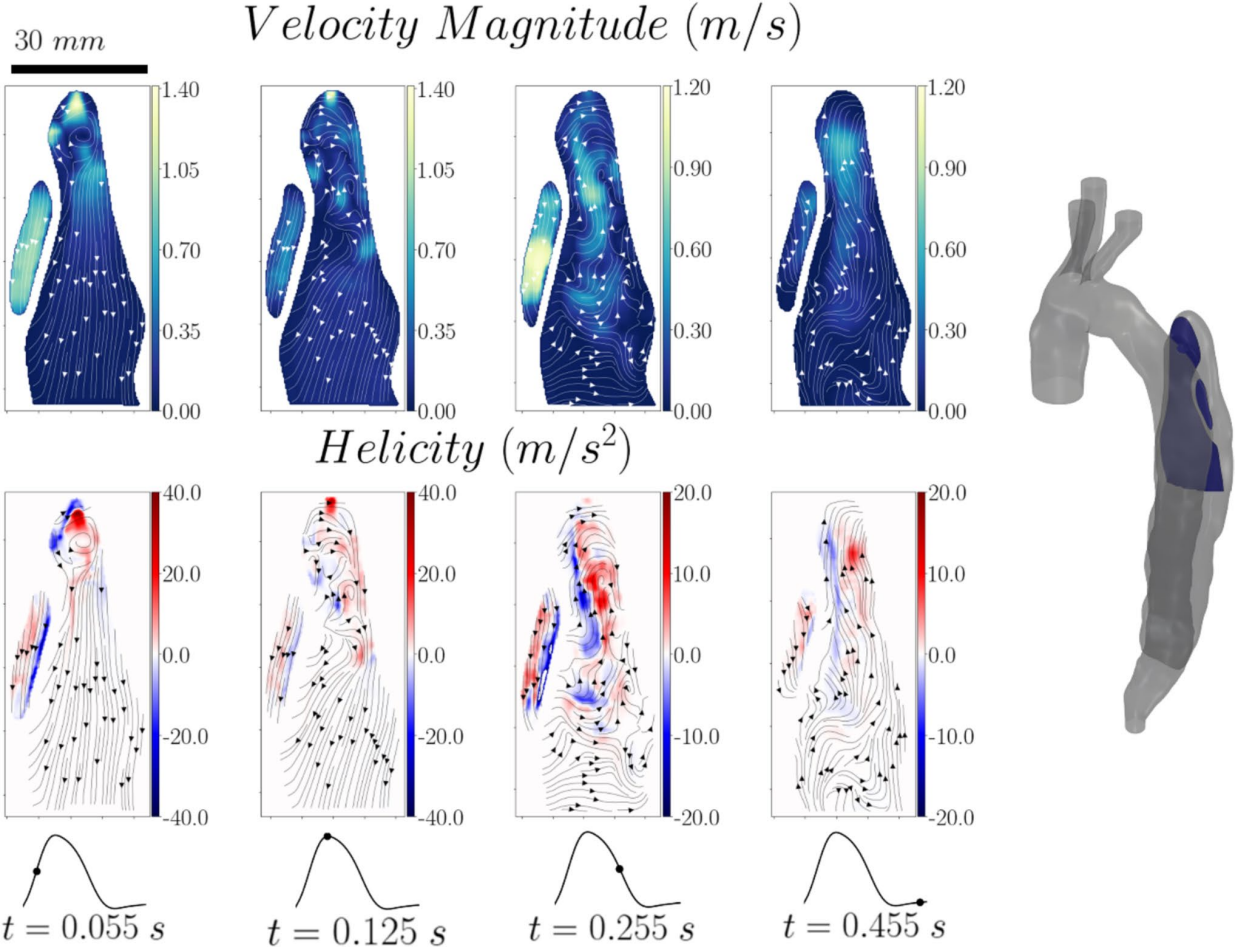
Velocity magnitude and helicity contour maps with superimposed streamlines for Plane 2

This flow behaviour can also be observed in the velocity and helicity contour plots of Plane 2, the second vertical plane through the proximal dissection imaged, perpendicular to Plane 1 (see Fig 4). In this case the systolic phase (*t* = 0.055 s and *t* = 0.125 s) is characterized by only a very localised high velocity region due to the fact that the axis of the generated jet is perpendicular to the measured plane.

However, the flow field on this plane highlights the transition of the flow from a more organised state during the systolic phase to a more chaotic one in the diastolic phase due to the aforementioned flow reversal. The extent of the high velocity region inside the false lumen is also clearly illustrated. Our previous in silico work based on a rigid wall assumption did not indicate substantial flow inside the false lumen, but our compliant simulations for the *same* patient did [5]. The presence of this region highlights the effect of wall compliance on the generated flow field inside the flow lumen and underscores the importance and relevance of compliance when trying to predict the haemodynamics of these complex, pathological aortas, which would have implications for prognosis. The extent of the non-stagnated flow region can also serve as a potential haemodynamic marker for partial or complete thrombosis of the FL.

**Fig. 5:**
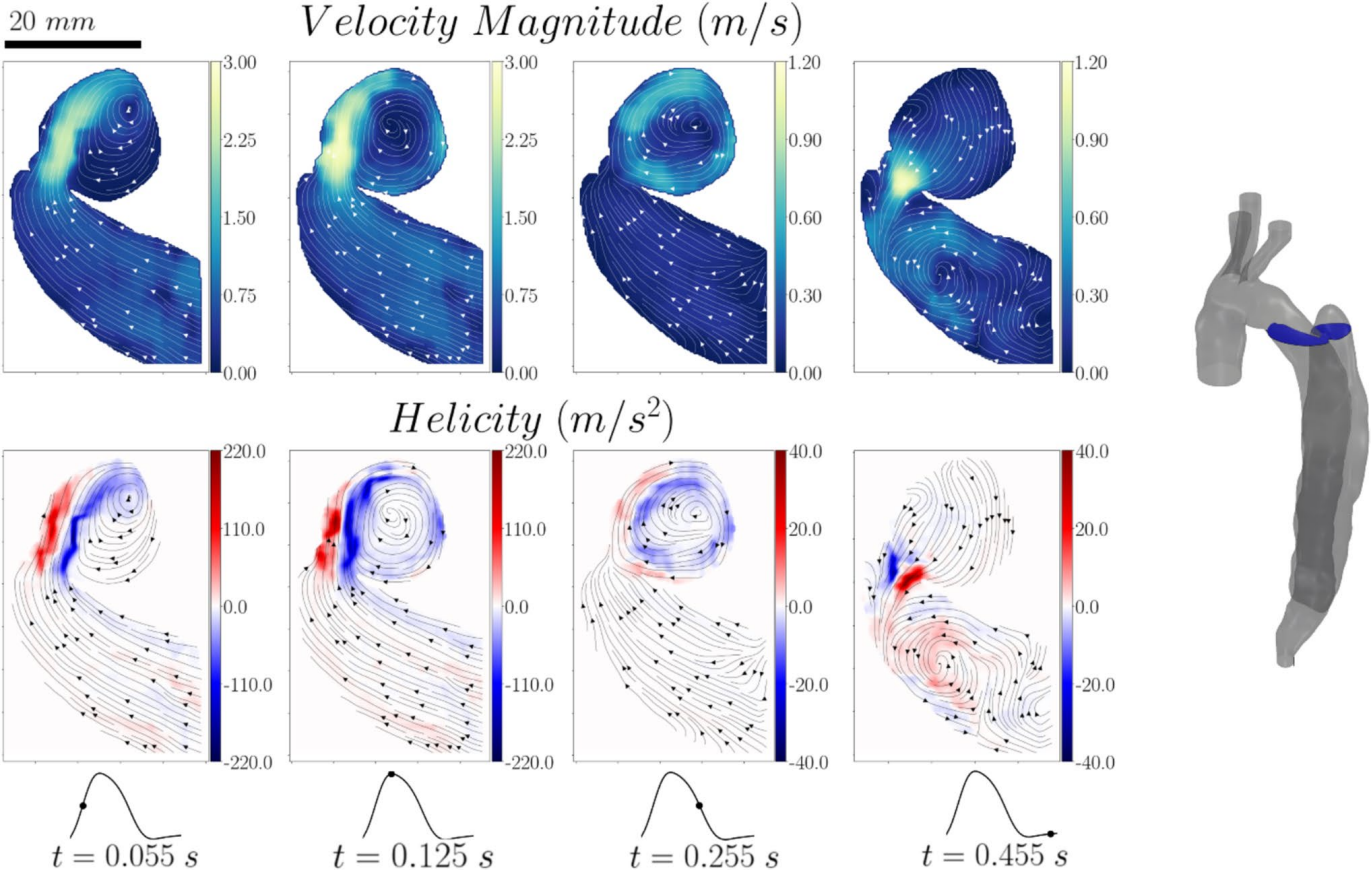
Velocity magnitude and helicity contour maps with superimposed streamlines for Plane 3

**Fig. 6:**
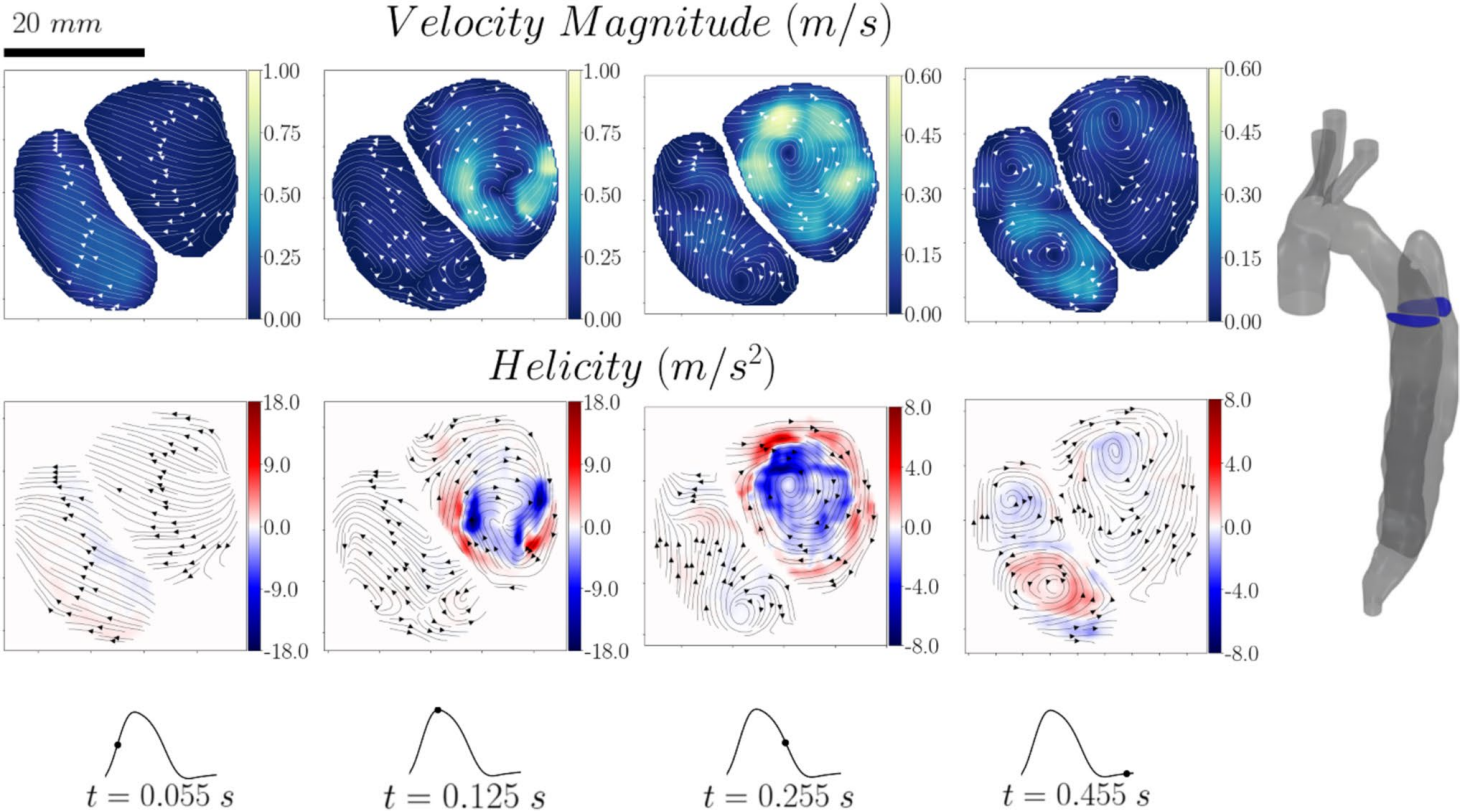
Velocity magnitude and helicity contour maps with superimposed streamlines for Plane 4

The flow field on two selected planes across the false lumen (Planes 3 and 4) is illustrated in Figs. 5 and 6, respectively. Plane 3 (see Fig 5) shows the flow field right at the entry tear of the false lumen. It clearly captures the jet entering the false lumen during the systolic phase and its impingement on the wall leading to the formation of a large vortical structure. During the diastolic phase we can observe the reversal of the flow with the jet re-entering the true lumen, generating two counter rotating vortices therein. Due to the small diameter of the entry tear, the jet velocities reach 4 and 2 times the one imposed at the inlet of the aortic arch, upon entering and exiting the false lumen, respectively. The velocities in the rest of the flow domain remain in the same order of magnitude to the inlet one. Plane 4 (see Fig 6) illustrates the flow field in a cross-sectional plane located 3 cm below the entry tear and Plane 3. The results show that the vortical structures induced near the entry tear propagate towards the medial part of the false lumen, leading to high velocities near the aortic walls. During both the systolic and early diastolic phase, the false lumen velocities remain in the same order of magnitude as the imposed inlet velocity. Late in diastole, two vortical structures are observed covering most of the true lumen cross section while rotational but low-velocity flow dominates the false lumen. These flow features in combination with the stagnant flow further downstream in FL might favour partial thrombosis and aneurysmal growth in this dissection. Clinical images indicate that partial thrombosis was evident in this patient [21].

**Fig. 7:**
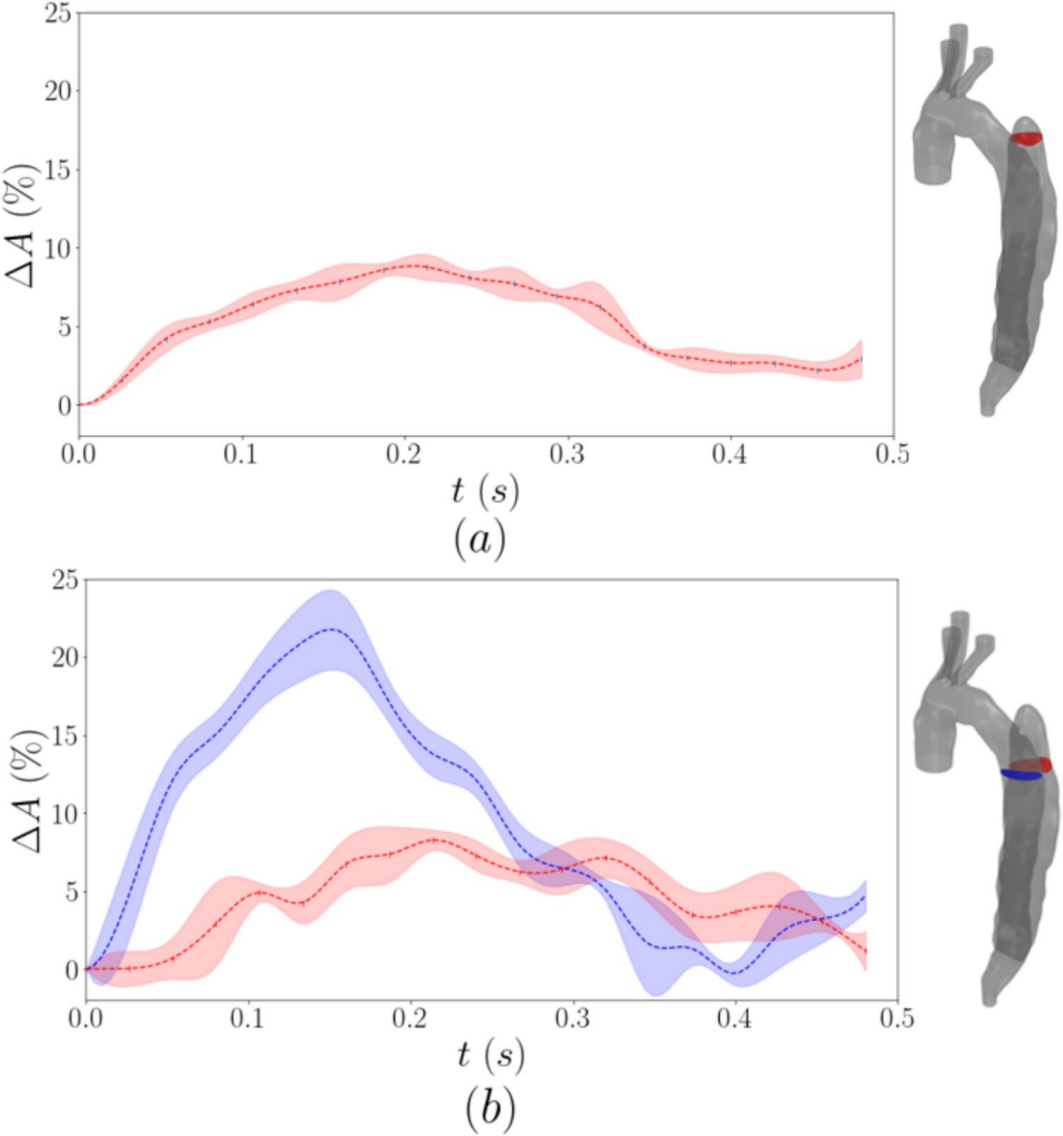
Cross-sectional area variation for the FL (red) in Plane 3 (**a**) and both the FL (red) and the TL (blue) in Plane 4 (**b**). The dotted line represents the mean values and the shaded area the standard deviation

The cross-sectional area variation over the cardiac cycle is illustrated in Fig 7 for Planes 3 and 4. Due to geometric constrictions, the boundaries of the true lumen in Plane 3 could not be well defined and thus its area variation is omitted. The FL exhibits a maximum area change of about 9% for both Planes 3 and 4, while the increase of the TL is about 22%. As expected, the maximum area change for the TL happens around the peak systole (*t* = 0.125 s).

On the contrary, the maximum wall deformation for the FL occurs in the deceleration and diastolic phases. This could be attributed to a gradual pressurisation of the false lumen due to the absence of an exit tear or other fenestrations. The area variation is similar in both imaged planes of the proximal false lumen in Fig 7a and b.

**Fig. 8:**
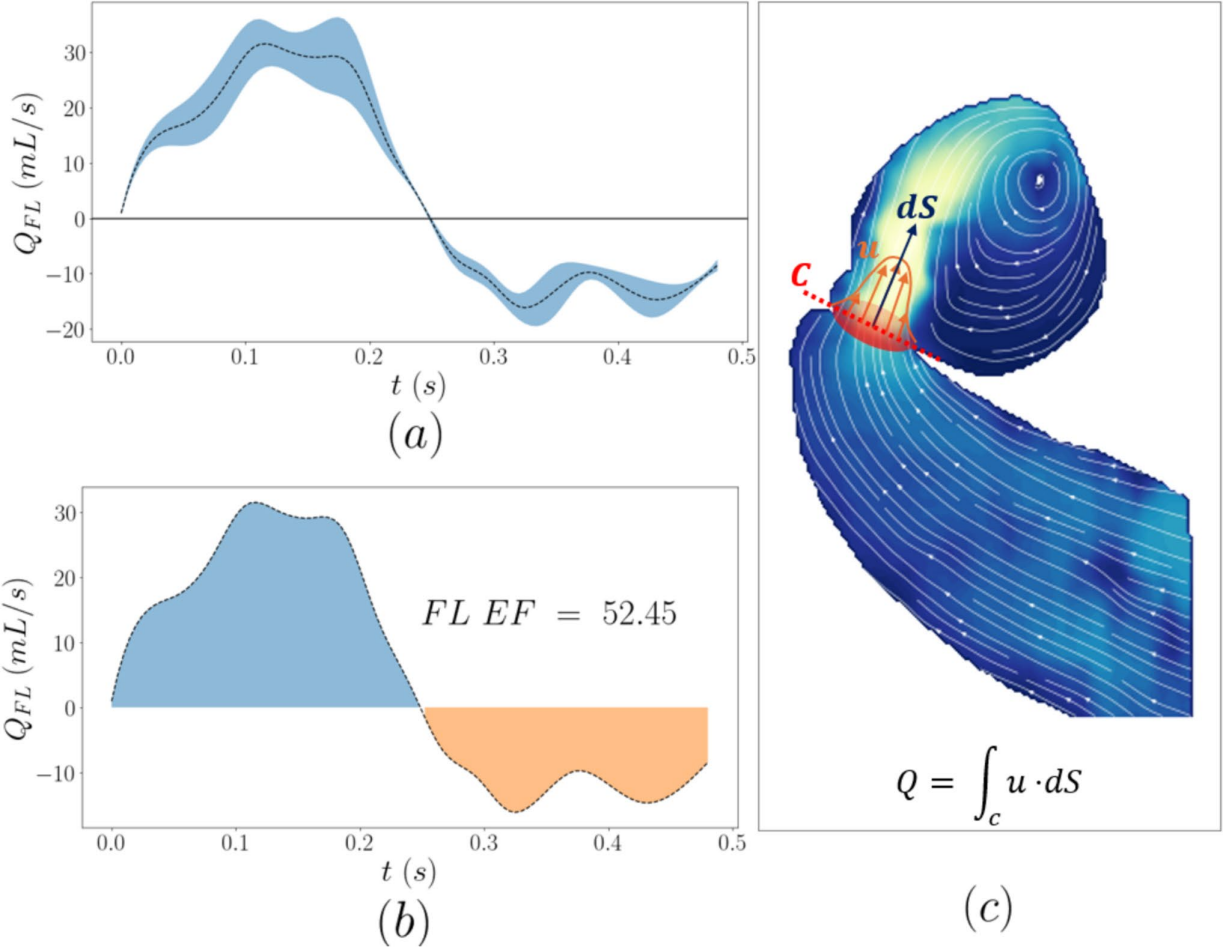
In-plane flow rate across the entry tear of the false lumen (**a**), false lumen ejection fraction calculation schematic (**b**), and flow rate integration schematic (**c**)

The in-plane flow rate at the entry tear as well as the false lumen ejection fraction (FLEF) are illustrated in Fig. 8. The in-plane flow rate was calculated by integrating the velocity profile at the entry tear assuming a circular cross-sectional area. The FLEF is defined as the ratio of retrograde flowrate during diastolic phase over the antegrade systolic flow rate. The calculated FLEF is 52.45% indicating a significant amount of retrograde flow at the entry tear. This index has been associated with aortic growth; elevated values have been found to correlate with enlarged dissections [25].

## Discussion

### Compliant Versus Rigid Wall Assumptions

The findings of this study emphasise the importance of the use of flexible phantoms for the investigation of false lumen haemodynamics as they are able to capture the dynamics of the wall motion and flow in the false lumen. A significant amount of flow was found to enter the false lumen resulting in a strong recirculation region in the proximal part, the extent of which might affect aneurysmal growth. The flow rate into the false lumen is in agreement with the compliant numerical simulations of [5] based on a moving boundary method. They predicted a mean flow rate of 11.8ml/s at systole and −8.7ml/s at diastole in the medial plane of the false lumen compared to 16.7ml/s and −13.8ml/s, respectively, measured at the entry tear in the present study. On the contrary no net flow into the false lumen was observed under a rigid wall assumption in the same study [5], whereas an in vitro study using a rigid phantom of the same AD [21] showed negligible levels of flow in the proximal false lumen. Velocities measured in a plane similar to Plane 2 in Fig 4 were less than < 0.05 m/s, i.e., an order of magnitude lower than the present study.

The cross-sectional area of the true lumen varied by up to 22% at peak systole whereas that in the false lumen varied up to 9%. The values are close to those predicted by [5], they computed a maximum percentage cross-sectional area variation of 10.7% and 17.2% at the ascending and abdominal aorta of this patient, whereas 2D-MRI data indicated 11.0% and 16.6%, respectively. The higher wall deformation recorded in the true lumen is the result of the highest pressure therein as indicated by the positive transmural pressures predicted by [5] throughout systole in a proximal section of the dissection. The transmural pressure was found to become negative in diastole with a minimum value of −10mm Hg (compared to 30mm Hg for systole), which can explain the lower wall deformation observed in the false lumen.

This study reinforces and expands upon our previous moving boundary simulations [5], highlighting the importance of accounting for wall motion to accurately estimate haemodynamic markers. Failure to do so could lead to misleading predictions regarding false lumen expansion or thrombosis, with important implications for patient outcomes. Our experimental results show strong qualitative agreement with prior simulations [5] based on clinical imaging data, particularly in terms of flow topology and wall motion patterns. These results highlight the potential of our framework to inform and validate computational models in future studies, particularly as we incorporate the measured wall deformations into the moving boundary simulations.

### False Lumen Flow as Predictor of Adverse Outcomes

The present study quantifies several parameters that have been identified as potential markers for aortic growth and complications in the literature. For example, the *in vivo* study of Evangelista et al. [26] indicates that the false lumen flow pattern observed in the present study might be a predictor of adverse outcomes. They correlated flow patterns observed in the false lumen by MRI with long-term outcomes in 131 AD patients. Their findings showed that a flow pattern characterised by large entry flow into the FL (above 30%), in the absence of similar distal discharge, has a high predictive value associated with faster enlargement rates and higher risk of complications. The authors found low aortic enlargement and few complications during follow-ups when the *high* antegrade FL flow was accompanied by low retrograde diastolic flow. On the contrary the presence of significant diastolic retrograde flow identified the subgroup of patients with the worst prognosis.

The false lumen ejection fraction was found to be 52.45% in the present study, indicating a balanced outflow–inflow in the FL. Interestingly, FLEF values of comparable magnitude were found to be associated with enlarged dissections in the 4D flow MR prospective study by Burris et al. [25]; an AD case with FL EF of 51% was shown to have grown by 4 mm over a 4-month period, a growth rate of 12 mm/year. The authors studied 18 patients with chronic TBAD and found a moderate-strong correlation between FLEF at the entry tear and aortic growth. It was suggested that this marker can serve as a risk predictor and also provide a measure of false lumen pressurisation, which cannot be determined in the clinic. No longitudinal data are available for the patient in the present study to verify this.

The same study [25] found that the peak velocity at the entry tear jet was lower in patients with enlarged FL. However, in the present study, a high peak velocity of almost 4 times that of the inlet velocity at the ascending aorta was found, which dropped to about 2 times at the outflow.

The impingement of the jet onto the opposite wall of the false lumen and the accompanying vortical structures lead to increased pressure and wall shear stress, potentially contributing to aortic growth and remodelling, which might end up compromising the structural integrity of the aortic wall. A recent paper by Baumler et al. [27] reports the simulation-based analysis performed on a rich dataset that shows the evolution of a Type B Aortic Dissection in a female patient. Simulations indicated that flow jet impingement in the false lumen resulted in a localised pressure increase of 11 and 2 mmHg in the subacute and chronic phases. These haemodynamic changes appear to be the main drivers of aortic growth and morphological changes when compared to the images acquired up to 64 months after disease onset.

It should be noted that the above markers, although estimated at the primary entry tear, depend very much on the number of fenestrations in the dissection which determines the false lumen flow characteristics. Additional intermediate fenestrations have been found to significantly reduce the false lumen flow reversal [13] which emphasises the need for a study of a range of AD morphologies to fully characterise FL haemodynamics.

False lumen haemodynamics have also been linked to thrombosis, in particular the presence of partial vs complete thrombosis as the former is associated with poor outcomes and high mortality. The false lumen flow fields in the present study indicate that despite the strong rotational flows in the upper part of the false lumen, stagnation flow is still present further downstream which might be evidence of partial thrombosis. This is due to the particular AD morphology studied here which is characterised by the absence of an exit tear or intermediate fenestrations. Clinical images for the present AD case indicated partial thrombosis. The longitudinal study by Ruiz-Muñoz et al. [28] based on 4D flow MR quantification of various haemodynamic markers, showed that partial thrombosis was associated with lower inflow ratio and kinetic energy and more stagnated flow. Out of all the flow markers examined, only the kinetic energy was found to be a main flow discriminator between the presence and absence of partial FL thrombosis and also independently be related to the extent of the thrombus. Interestingly, the strength of the rotational flow could not differentiate between the thrombosed and partially thrombosed groups.

### Limitations

The study presents the first attempt to experimentally characterise the patient-specific AD false lumen flows with Particle Image Velocimetry using a flexible phantom and is subject to some limitations. Due to the delicate nature of the flexible phantom, and the continuous pulsatile nature of the flow the number of consecutive cycles that could be acquired was small. We tried to partially compensate for this by increasing the temporal resolution within the cycle. A 2D-PIV method and a Newtonian blood analog were employed to characterize the flow field. Knowledge of the third velocity component would have allowed more accurate estimation of haemodynamic markers, particularly for the case of chaotic flows such the ones reported for the diastolic phase. A non-Newtonian fluid would also be more realistic and this will be the subject of future work. However, in our previous work with a rigid AD geometry [19, 21] we studied the influence of blood rheology on flow predictions and the assumption of Newtonian rheology was deemed acceptable. In the present work the aortic phantom was surrounded by the refractive index matched fluid whose properties differ from the biological tissue surrounding the aorta. This posed challenges in the selection of measured planes and might limit the physiological relevance of the estimated wall response. Future work will address this limitation.

The study makes use of one patient-specific TBAD geometry with one primary entry tear. To fully understand the complex FL flows and extract useful markers for aortic growth and risk prediction more patient geometries with an exit as well as intermediate tears should be interrogated as evidenced by 4D flow MR studies. Nevertheless, the present work demonstrates the potential of this in vitro approach in capturing false lumen flows with unprecedented levels of resolution and detail that is not possible with clinical modalities. Such approaches can serve as validation tools for computational simulations; they can also be combined with in vitro 4D flow-MRI measurements to better understand AD flows and to aid the further development of haemodynamic markers that can be used in clinical practice.

## Conclusion

The present work investigated the false lumen haemodynamics in a Type B aortic dissection using a patient-specific flexible phantom and particle image velocimetry The measured flow fields allowed markers such as helicity, the false lumen ejection fraction and the true and false lumen area changes during the cardiac cycle to be determined and compared with the existing literature. The results highlight the importance of wall compliance on the flow field inside the false lumen that cannot be reproduced experimentally or computationally under the assumption of rigid walls. The false lumen markers seem to agree with clinical studies, and in combination with the calculated flow fields provide a better understanding of both their physical and clinical impact. Future studies should focus on using similar approaches on other patient-specific geometries with systematic comparison between rigid and compliant phantoms to aid further understanding of the haemodynamic phenomena of AD and refine computational predictive tools. A combination of these measurements with in vitro studies using medical imaging modalities (e.g. 4D flow-MRI) could provide the clinician with valuable information to help in the planning of interventions and predict patient outcomes.
